# A new peritoneal dialysis fluid for Japanese patients: a randomized non-inferiority clinical trial of safety and efficacy

**DOI:** 10.1007/s10157-016-1346-9

**Published:** 2016-10-25

**Authors:** Masaaki Nakayama, Yoshindo Kawaguchi, Takashi Akiba, Masao Kim, Hidemune Naito, Shigeko Hara, Teruhiko Maeba, Noriaki Yorioka, James A. Sloand, Mark R. Marshall

**Affiliations:** 1 Department of Nephrology and Hypertension, School of Medicine, Fukushima Medical University, Fukushima, Japan; 2 Department of Internal Medicine, Nephrology and Hypertension, Tokyo Jikei-kai Medical School, Tokyo, Japan; 3 Hospital Affiliating with Kanagawa Prefecture Nursing School, Isehara, Kanagawa Japan; 4 Sekikawa Hospital, Tokyo, Japan; 5 Department of Blood Purification, Tokyo Women’s Medical University, Tokyo, Japan; 6 Kaikou Clinic, Osaka, Japan; 7 Osaka Dialysis Department, Kaiko-Kai Clinic, Osaka, Japan; 8 Naito Medical Research Laboratory, Kobe, Hyogo Japan; 9 Hakubi-kai, Sano Ikawadani Hospital, Kobe, Hyogo Japan; 10 Hara Press Center Clinic, Tokyo, Japan; 11 Kidney Center, Toranomon Hospital, Tokyo, Japan; 12 Asao Kidney Clinic, Kawaski, Kanagawa Japan; 13 Department of Internal Medicine, Nephrology and Hypertension, St. Marianna University School of Medicine, Kawaski, Kanagawa Japan; 14 Hiroshima Kidney Organization, Hiroshima, Japan; 15 Department of Molecular and Internal Medicine, Graduate School of Biomedical Sciences, Hiroshima University, Hiroshima, Japan; 16 Therapeutic Area, Baxter Healthcare Corporation, Deerfield, IL USA; 17 Therapeutic Area, Baxter Healthcare (Asia) Pte Ltd, Singapore, Singapore; 18 School of Medicine, Faculty of Medical and Health Sciences, University of Auckland, Parnell, PO Box 37968, Auckland, 1151 New Zealand

**Keywords:** Peritoneal dialysis, Metabolic alkalosis, Dialysis adequacy, Biocompatibility

## Abstract

**Background:**

We report here two new peritoneal dialysis fluids (PDFs) for Japan [BLR 250, BLR 350 (Baxter Limited, Japan)]. The PDFs use two-chamber systems, and have bicarbonate and lactate buffer to a total of 35 mmol/L. In separate trials, the new PDFs were compared to two “standard” systems [PD-4, PD-2 (Baxter Limited, Japan)]. The trials aimed to demonstrate non-inferiority of peritoneal creatinine clearance (pCcr), peritoneal urea clearance (pCurea) and ultrafiltration volume (UF), and compare acid–base and electrolyte balance.

**Methods:**

We performed randomized, multicenter, parallel group, controlled, open-label clinical trials in stable continuous ambulatory peritoneal dialysis (CAPD) patients. The primary endpoints were pCcr and UF. The secondary endpoints were serum bicarbonate and peritoneal urea clearance. The active phase was 8 weeks. These trials were performed as non-inferiority studies, with the lower limit of non-inferiority for pCcr and UF set at 3.2 L/week/1.73 m^2^ and 0.12 L/day, respectively.

**Results:**

108 patients (28 centers) and 103 patients (29 centers) took part in the two trials. Groups were well balanced at baseline. The investigative PDFs were non-inferior to the “standard” ones in terms of primary endpoints, comparable in terms of pCurea, and superior in terms acid–base balance, especially correcting those with over-alkalinization at baseline.

**Conclusions:**

We demonstrated fundamental functionality of two new PDFs and showed superior acid–base balance. Given the propensity of Japanese CAPD patients for alkalosis, it is important to avoid metabolic alkalosis which is associated with increased cardiovascular mortality risk and accelerated vascular calcification. The new PDFs are important progress of CAPD treatment for Japanese patients.

**Electronic supplementary material:**

The online version of this article (doi:10.1007/s10157-016-1346-9) contains supplementary material, which is available to authorized users.

## Introduction

There is global interest in expanded use of peritoneal dialysis (PD) to improve clinical outcomes and meet increasing resource constraints. In Japan, the limited availability of kidney transplantation means that dialysis is a lifelong necessity for most patients with end-stage kidney disease. As a result, vintage on PD therapy in Japan is longer than that in either the US or European countries. This prolonged exposure to PD fluid has been of major clinical concern in those countries, due to the untoward cellular, local, and systemic effects from un-physiologic components within them. High glucose concentration, glucose degradation products (GDPs), lactate, high osmolality, and low pH are all accepted as un-physiologic factors, with potential for harm to both the peritoneal membrane as well as the systemic milieu [1–10].

The biocompatibility of PD fluids (PDFs) can be increased through the use of a multi-compartment bag system that separates out the buffer from the glucose. Heat sterilization and storage occur at a low pH, minimizing glucose degradation and formation of GDPs. In addition, the system allows the use of a bicarbonate buffer, without the risk of precipitation with magnesium and calcium. After mixing of compartments, the solution has a neutral or close to neutral pH, with relatively low GDP and lactate content. The first system using these new PDFs (Physioneal®, Baxter Healthcare Inc, containing bicarbonate 25 mmol/L and lactate 15 mmol/L) was developed approximately 20 years ago and continues to be available in many parts of the world today. [11–13].

However, new PDFs with all these properties have not been commercially available until recently in Japan. Previously, there was an assessment of a two-chamber bag system containing 25 mmol of bicarbonate with 15 mmol of lactate as buffer (Physioneal-40^**®**^, Baxter Healthcare, Deerfield, IL). A clinical trial was performed in 1997 and showed increased metabolic alkalosis (plasma bicarbonate 29.8 mmol/L) in comparison to control PDF (plasma bicarbonate 28.2 mmol/L) [14]. Although there were no accompanying clinical symptoms, there was much concern that Physioneal-40 would over-correct metabolic acidosis (i.e., causing alkalosis) in Japanese patients.

In this paper, we present the first definitive testing of two new PDFs [BLR 250, BLR 350 (Baxter Limited, Japan)], which have recently been made available for Japanese patients. The new PDFs use a twin-bag two-chamber system for enhanced biocompatibility, with buffer consisting of a mixture of bicarbonate and lactate and a total alkali content of 35 mmol/L (bicarbonate 25 mmol/L and lactate 10 mmol/L). In separate trials, the two new PDFs were compared with the standard twin-bag single-chamber system for efficacy and safety. The main aim of both trials was to ensure non-inferiority of delivered dialysis dose (pCcr) and ultrafiltration (UF) in comparison to the standard PDFs, and compare acid–base and electrolyte balance.

## Methods

### Study design

The trials for BLR 250 and BLR 350 were conducted separately, but were identical in design: randomized, parallel group, controlled clinical trials of investigative PDFs vs. standard PDFs. The trials are registered with the ISRCTN registry (ISRCTN #10007426 and ISRCTN#48112900).

Participants were recruited from dialysis centers across Japan. Research staff within each dialysis center performed recruitment upon referral of patients after an assessment by the usual clinical team.

**Fig. 1:**
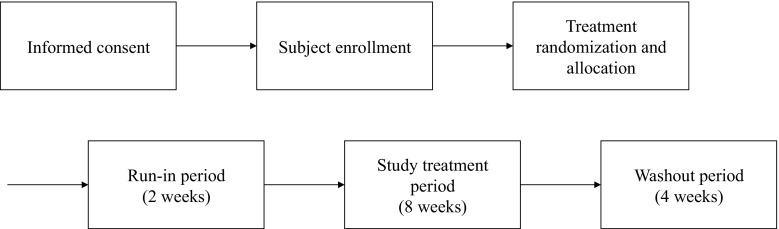
Study design of the BLR 250 and BLR 350 trials

All participants underwent a run-in period of 2 weeks using the standard PDFs before the active trial period, during which their usual PD prescription and PDFs were applied. This was followed by active trial period of 8 weeks during which they received either investigative or standard PDFs. Finally, all participants underwent a washout period of 4 weeks during which participants returned to their usual PD prescription and PDFs (Fig. 1).

The trials were conducted in accordance with the good clinical practice (GCP) guidelines and the Declaration of Helsinki. Prior to study initiation, ethics approvals were obtained from Institutional Review Boards of each trial site. All participants provided full written informed consent. Patient recruitment, randomization, and allocation were managed by EPS Co., Ltd (Shinjuku-ku, Tokyo, 162-0822, Japan). Data collection and entry was managed by Quintiles Transnational Japan (Minato-ku, Tokyo, 108-0074, Japan). Data audit and statistical analysis were by Bell System 24, Inc. (Chuo-ku, Tokyo, 104-6113, Japan).

### Participant eligibility

Adult patients aged 20 years or older were eligible to participate. Inclusion criteria were: end-stage kidney failure treated with maintenance CAPD for at least 3 months prior to enrollment; treatment with the Dianeal^®^ PD system (Baxter Limited, Japan) with 2L exchanges 3–5 times a day for at least 4 weeks prior to enrollment. Exclusion criteria were: active malignancy; acute or chronic liver disease; chronic heart failure; active systemic infection; severe malnutrition; known peritoneal dysfunction (high transporter); peritonitis within the 4 weeks prior to enrollment; and high likelihood converting to hemodialysis (HD). Patients who were on Dianeal PD-4^®^ (calcium concentration 1.25 mmol/L) prior to the study were eligible for the BLR 250 trial; those on Dianeal PD-2^®^ (calcium concentration 1.75 mmol/L) prior to the study were eligible for the BLR 350 trial.

### Interventions

The investigative PDFs were both provided in twin-bag two-chamber system that was mixed just before use by patient breakage of the inter-chamber seal. Both investigative PDFs were characterized by 25 mM of bicarbonate and 10 mM of lactate. BLR 250 had a lower calcium concentration (1.25 mmol/L), and the BLR 350 had a higher calcium concentration (1.75 mmol/L).

In the BLR 250 trial, participants randomized to either the investigative PDF or their standard Dianeal PD-4^®^. In the BLR 350 trial, participants randomized to either the investigative PDF or their usual Dianeal PD-2^®^. All participants continued their standard CAPD prescription of between three to five 2L bag exchanges per day, continued without change of bag number throughout the study period from the baseline period through the follow-up period. The detailed composition of the investigative and standard PDFs is summarized in Table 1.

**Table 1:** Composition of the investigative and standard peritoneal dialysis fluids (PDFs)

Composition	BLR 250			Dianeal^®^ PD-4			BLR 350			Dianeal^®^ PD-2		
	1.5	2.5	4.25	1.5	2.5	4.25	1.5	2.5	4.25	1.5	2.5	4.25
Glucose (anhydrous) (%)	1.36	2.27	3.86	1.36	2.27	3.86	1.36	2.27	3.86	1.36	2.27	3.86
Na^+^ (mmol/L)	132			132			132			132		
Ca^2+^ (mmol/L)	1.25			1.25			1.75			1.75		
Mg^2+^ (mmol/L)	0.5			0.5			0.5			0.5		
Cl^−^ (mmol/L)	100			95			101			96		
Lactate (mmol/L)	10			40			10			40		
HCO_3_^−^ (mmol/L)	25			–			25			–		
pH	6.8–7.8			4.5–5.5			6.8–7.8			4.5–5.5		
Osmolarity (mOsm/L)	344	395	483	344	395	483	346	396	484	346	396	485

### Outcomes

Study outcomes were the same in both trials. The primary outcomes were pCcr (L/week/1.73 m^2^) and UF (L/day). pCcr and UF were assessed from 24-h collections of dialysate (accounting for residual renal function if urine volume was greater than 100 mL/day). Baseline pCcr (L/week/1.73 m^2^) and UF (L/day) were reported as the average of measurements at the beginning and end of the run-in period. Measurements were repeated every 4 weeks during the active period. pCcr (L/week/1.73 m^2^) was calculated by the following formula:

$$\frac{D \times V}{P} \times 7 \times \frac{1.73}{X},$$

where *D* is the creatinine concentration in the effluent, *P* is the creatinine concentration in the plasma, *V* is the 1-day effluent volume (L/day), and *X* is the area of body surface (m^2^) [= 0.007184 × height (cm)^0.725^ × body weight (kg)^0.425^].

UF was calculated by subtracting the total infused volume from the total volume of effluent (corrected to the 24-h rate).

The use of two primary endpoints raises the issue of multiplicity. To avoid this issue, we gave priority to pCcr as an indication of the efficacy. Of note, the formula to calculate pCcr also includes the volume of ultrafiltration, showing that UF partially considered in the calculation of pCcr. Priority was thus given to pCcr.

The secondary outcomes were pCurea per week (weekly Kt/Vurea) and plasma bicarbonate concentration, all measured at the same time-points and reported in the same manner as the primary outcomes. pCurea was calculated by the same formula as pCcr using corresponding data for urea rather than creatinine.

Biochemical parameters other than plasma bicarbonate were measured at BML Co. Ltd. (Shibuya-ku, Tokyo, 151-0051, Japan), an external central laboratory. Acid–base status was evaluated by measuring pH and pCO_2_ in venous blood sampled from the antecubital fossa using a conventional blood gas analyzer at each institution. The procedure for measurement was determined according to the one used for blood gas measurement performed as part of routine clinical care at the respective institution, and measurement performed accordingly. Bicarbonate concentrations were calculated from Henderson–Hasselback equation.

### Sample size, randomization, and blinding

Power was determined with an 80% power to test the null hypothesis of non-inferiority when the alternative hypothesis of inferiority is true, at a one-sided alpha level of 0.025. Inferiority was defined by a clinically significant relative decrease greater than 3.2 l/week/1.73 m^2^ for pCcr, and by a corresponding decrease of 0.12 L/day for UF. The rationale for the limits of non-inferiority was as follows. In the clinical study with Physioneal-40^®^ performed formerly in Japan [15], the standard deviations of change from baseline in pCcr and UF were 6.3 (L/week/1.73 m^2^) and 0.241 (L/day), respectively, in subjects treated with Dianeal PD-4^®^. We considered that half of the standard deviation would be a clinically acceptable range of non-inferiority.

For calculation of the sample size under a non-inferiority framework, we used a one-sided significance level (*α*) of 2.5% and the power (1−*β*) of 80%. Since the variables to assess non-inferiority were to be measured twice, namely at weeks 4 and 8, we used the mean of both changes for analysis to minimize the error variance for calculations. The correlation coefficient between the two values (changes at weeks 4 and 8) was assumed to be approximately 0.45 for each variable. The necessary number of patients per group was calculated to be 46 by the equation below. Hence, we decided to enroll 100 patients, considering possible exclusions and discontinuations/dropouts

$$n = \left[ {2 \times \left( {z_{\alpha /2} + z_{\beta } } \right)^{2} \left( {\frac{\sigma }{\Delta }} \right)^{2} \left( {\frac{1 + \rho }{2}} \right) + 1} \right],$$

where [] is the Gauss notation, *z* is the upside percent point of standard normal distribution, *σ* is the standard deviation, Δ is the margin of non-inferiority, and *ρ* is the correlation coefficient.

Randomization was made by a third-partly clinical research organization using a computer-generated sequence. Allocation was made in a 1:1 ratio in blocks of 4 patients (by study site), concealed from investigators, patients, and the sponsor (except for the Study Drug Quality Control Manager) until the completion of the run-in period. The study was single-blinded (outcomes assessor), without blinding of patients or clinical staff.

### Statistical methods

Where necessary, descriptive statistics used Fischer’s exact test for non-parametric measures and Student’s *t* test for parametric ones. A *p* value of 0.05 or less was considered statistically significant.

Given the non-inferiority framework of the study hypotheses, we performed statistical analyses of the primary outcomes in both intention-to-treat (ITT) and per-protocol (PP) subsets of the study population. The ITT subset was defined as all participants randomly allocated to treatment groups, who received a study treatment at least once, and had no violations of selection criteria or serious (e.g., GCP) violations in the run-in period. The PP subset excluded participants under the following conditions: missing efficacy data, major protocol violations, including crossovers, severe lack of compliance, and where conditions were discovered a posteriori not to have been met. For secondary and ancillary outcomes, we performed analyses on the ITT subset only.

For the primary and secondary outcomes, we analyzed mean changes at weeks 4 and 8 from baseline. Analysis of covariance (ANCOVA) was performed using baseline values as covariates, mean values of changes at weeks 4 and 8 from baseline as objective variables, and the treatment groups as factors. Based on ANCOVA without interaction between the treatment groups and covariates, the 95% confidence intervals of the adjusted mean differences between the treatment groups (the investigative PDF—the standard PDF) were calculated. If the lower limits in both ITT and PP analyses were not below the lower limit level of the non-inferiority [3.2 (L/week/1.73 m^2^) and 0.12 (L/day)] for pCcr and UF, respectively, the efficacy of the investigative PDFs was to be concluded to be not inferior to that of the standard PDF. Given the non-inferiority framework of the study hypotheses, a one-sided *p* value of 0.025 or less considered as statistically significant for these analyses.

Results were expressed as mean ± SD unless otherwise stated. All statistical analyses were performed using the SAS statistical software version 8.2 (SAS Institute, Japan).

## Results

### Participant flow

**Fig. 2:**
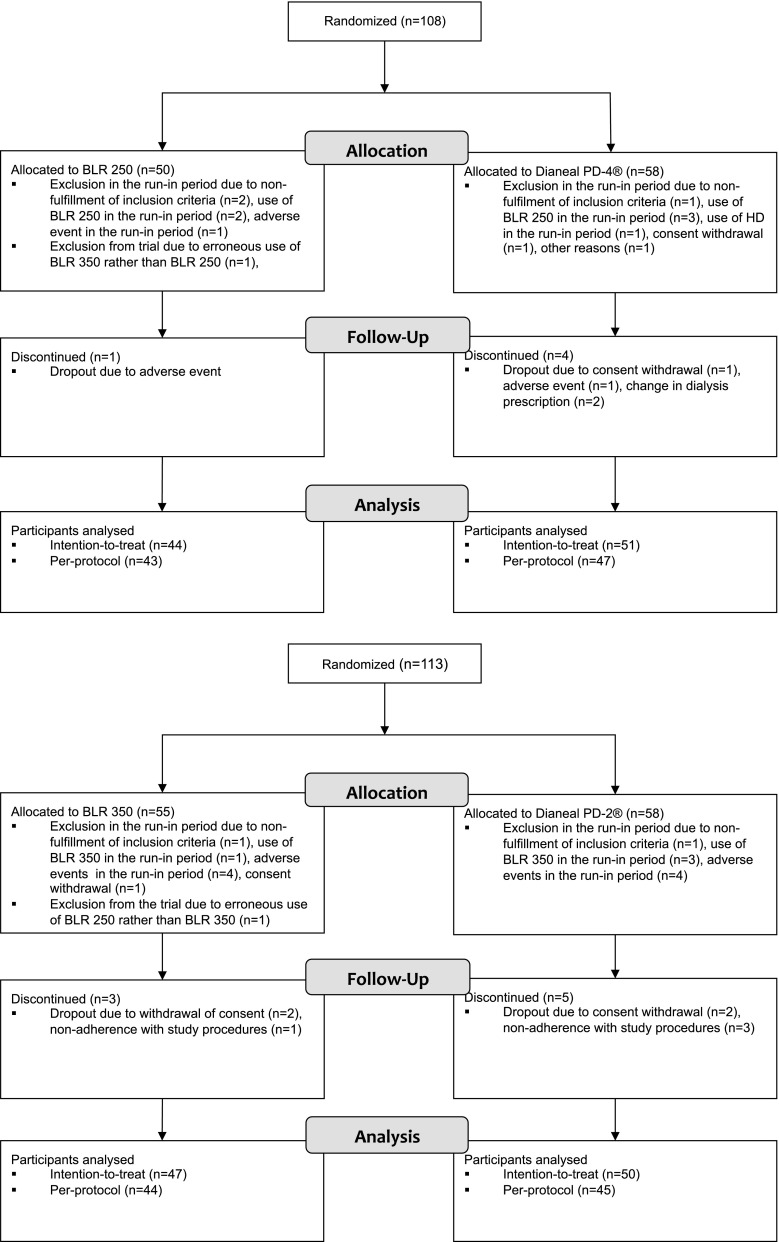
CONSORT 2010 participant flow diagrams for the BLR 250 trial (*top panel*) and BLR 350 trial (*bottom panel*)

Recruitment of patients for the BLR 250 trial was performed from March 24, 2003, with last patient follow-up to March 18, 2004, from a total of 28 participating sites (see on-line supplementary Appendix 2). Corresponding dates for the BLR 350 trial were November 6, 2002 and April 15, 2004, from a total of 29 participating sites (see on-line supplementary Appendix 2). The participant flow for both trials is shown in Fig. 2.

### Characteristics of patients who were eligible for participation

Table 2 shows baseline clinical characteristics of the participants in the BLR 250 and BLR 350 trials. There was no statistically and clinically significant imbalance between those receiving investigative or standard solutions arms in either trial, either for the ITT populations or for the PP populations. The only statistically significant difference was in the BLR 250 trial, where those who were randomized to the investigative PDF were more likely to have started with hemodialysis (HD) as the initial dialysis modality than those randomized to the standard PDF. Despite this, both groups had similar vintage and time on PD, and the effect of this imbalance on outcomes is likely to be negligible.

**Table 2:** Clinical characteristics of the intention-to-treat and per-protocol populations of the BLR 250 and BLR 350 trials

		Intention-to-treat population						Per-protocol population					
		BLR250	PD-4	*p* value	BLR350	PD-2	*p* value	BLR250	PD-4	*p* value	BLR350	PD-2	*p* value
*n*		44	51		47	50		43	47		44	45	
Gender	Male	32 (72.7%)	31 (60.8%)	0.28	38 (80.9%)	40 (80.0%)	1.00	32 (74.4%)	30 (63.8%)	0.36	35 (79.5%)	37 (82.2%)	0.79
	Female	12 (27.3%)	20 (39.2%)		9 (19.1%)	10 (20.0%)		11 (25.6%)	17 (36.2%)		9 (20.5%)	8 (17.8%)	
Age	Years	55.4 (12.5)	53.5 (12.9)	0.48	54.5 (13.0)	54.8 (9.8)	0.90	55.2 (12.6)	54.6 (12.0)	0.81	55.0 (13.2)	54.7 (9.3)	0.91
Height	cm	161.67 (7.11)	160.68 (8.80)	0.56	165.31 (9.25)	164.69 (7.74)	0.72	161.80 (7.13)	160.75 (8.57)	0.53	164.81 (9.14)	165.26 (7.49)	0.80
Weight	kg	59.48 (8.25)	58.74 (10.03)	0.70	65.53 (11.55)	63.20 (10.30)	0.30	59.36 (8.31)	58.50 (9.25)	0.65	65.32 (11.87)	64.29 (9.01)	0.65
Primary renal disease	Chronic glomerulonephritis	28 (63.6%)	33 (64.7%)	0.40	23 (48.9%)	29 (58.0%)	0.63	27 (62.8%)	32 (68.1%)	0.35	22 (50.0%)	27 (60.0%)	0.52
	Nephrosclerosis	3 (6.8%)	3 (5.9%)		2 (4.3%)	3 (6.0%)		3 (7.0%)	2 (4.3%)		2 (4.5%)	3 (6.7%)	
	Diabetic nephropathy	6 (13.6%)	2 (3.9%)		11 (23.4%)	6 (12.0%)		6 (14.0%)	2 (4.3%)		10 (22.7%)	5 (11.1%)	
	Other	2 (4.5%)	6 (11.8%)		5 (10.6%)	7 (14.0%)		2 (4.7%)	6 (12.8%)		4 (9.1%)	6 (13.3%)	
	Unknown	5 (11.4%)	7 (13.7%)		6 (12.8%)	5 (10.0%)		5 (11.6%)	5 (10.6%)		6 (13.6%)	4 (8.9%)	
Vintage	Months	64.4 (46.9)	60.7 (55.3)	0.74	36.3 (38.1)	37.6 (37.7)	0.87	62.7 (46.0)	61.9 (55.7)	0.95	36.3 (39.0)	34.9 (38.1)	0.87
Duration on PD	Months	54.6 (44.5)	54.9 (46.6)	0.97	31.1 (32.5)	35.2 (36.2)	0.56	54.2 (45.0)	55.8 (46.5)	0.88	30.7 (33.0)	32.2 (36.2)	0.84
History of Tx		1 (2.3%)	1 (2.0%)	1.00	1 (2.1%)	0 (0.0%)	0.49	1 (2.3%)	1 (2.1%)	1.00	1 (2.3%)	0 (0.0%)	0.49
Initial modality of RRT	CAPD	23 (52.3%)	39 (76.5%)	0.02	31 (66.0%)	38 (76.0%)	0.37	23 (53.5%)	37 (78.7%)	0.02	30 (68.2%)	34 (75.6%)	0.49
	IPD	2 (4.5%)	0 (0.0%)		0 (0.0%)	0 (0.0%)		2 (4.7%)	0 (0.0%)		0 (0.0%)	0 (0.0%)	
	HD	18 (40.9%)	12 (23.5%)		16 (34.0%)	12 (24.0%)		17 (39.5%)	10 (21.3%)		14 (31.8%)	11 (24.4%)	
	Other	1 (2.3%)	0 (0.0%)		0 (0.0%)	0 (0.0%)		1 (2.3%)	0 (0.0%)		0 (0.0%)	0 (0.0%)	
Peritonitis	Episode or episodes in last 6 months	1 (2.3%)	3 (5.9%)	0.62	2 (4.3%)	3 (6.0%)	1.00	1 (2.3%)	2 (4.3%)	1.00	2 (4.5%)	2 (4.4%)	1.00
Tunnel infection		0 (0.0%)	1 (2.0%)	1.00	1 (2.1%)	0 (0.0%)	0.49	0 (0.0%)	1 (2.1%)	1.00	1 (2.3%)	0 (0.0%)	0.49
Exit site infection		5 (11.4%)	11 (21.6%)	0.27	12 (25.5%)	7 (14.0%)	0.20	5 (11.6%)	10 (21.3%)	0.27	10 (22.7%)	6 (13.3%)	0.28
Peritoneal creatinine clearance	L/week/1.73 m^2^	53.29 (8.48)	56.41 (9.76)	0.10	50.82 (9.03)	50.10 (9.67)	0.71	53.32 (8.57)	56.61 (9.44)	0.09	50.92 (9.24)	49.47 (9.75)	0.47
Ultrafiltration volume	L/day	0.698 (0.524)	0.692 (0.548)	0.96	0.602 (0.467)	0.597 (0.391)	0.93	0.676 (0.508)	0.688 (0.516)	0.92	0.603 (0.455)	0.586 (0.384)	0.84
Peritoneal urea clearance	Week Kt/V	1.79 (0.29)	1.88 (0.36)	0.20	1.67 (0.33)	1.70 (0.33)	0.69	1.78 (0.30)	1.89 (0.37)	0.16	1.69 (0.33)	1.68 (0.31)	0.95
nPNA	g/kg/day	1.05 (0.17)	1.02 (0.19)	0.57	1.05 (0.17)	1.03 (0.19)	0.75	1.05 (0.18)	1.07 (0.14)	0.62	1.05 (0.18)	1.07 (0.14)	0.52
Plasma [HCO_3_^–^]	mmol/L	29.62 (2.72)	29.28 (2.69)	0.54	28.55 (2.63)	28.25 (2.95)	0.60	29.66 (2.74)	29.26 (2.79)	0.49	28.58 (2.67)	28.20 (2.92)	0.53
Serum [Na^−+^]	mmol/L	137.5 (3.4)	138.3 (4.1)	0.32	138.4 (3.3)	139.3 (3.2)	0.19	137.5 (3.4)	138.4 (4.1)	0.27	138.5 (3.3)	139.3 (3.3)	0.25
Serum [K^−+^]	mmol/L	4.08 (0.72)	3.83 (0.66)	0.09	4.17 (0.65)	3.97 (0.66)	0.15	4.04 (0.70)	3.85 (0.68)	0.19	4.14 (0.62)	4.00 (0.64)	0.28
Serum [Cl^−^]	mmol/L	95.1 (3.3)	95.6 (3.6)	0.49	96.3 (4.0)	97.8 (4.1)	0.08	95.1 (3.3)	95.7 (3.6)	0.43	96.2 (3.8)	97.9 (4.3)	0.06
Serum [Ca^2+^]	mmol/L	2.33 (0.23)	2.38 (0.27)	0.34	2.39 (0.24)	2.33 (0.18)	0.14	2.32 (0.22)	2.36 (0.27)	0.40	2.41 (0.23)	2.32 (0.19)	0.07
Serum [Mg^2+^]	mmol/L	0.90 (0.18)	0.84 (0.14)	0.07	0.91 (0.16)	0.87 (0.14)	0.26	0.90 (0.18)	0.84 (0.14)	0.11	0.91 (0.16)	0.87 (0.14)	0.36
Serum [PO_4_^2+^]	mmol/L	1.65 (0.40)	1.64 (0.40)	0.89	1.63 (0.39)	1.61 (0.38)	0.69 (0.40)	1.65 (0.40)	1.62 (0.39)	0.69	1.63 (0.39)	1.60 (0.39)	0.71

Results are presented as either *n* (%), or mean (standard deviation)

*CAPD* continuous ambulatory peritoneal dialysis; *IPD* intermittent peritoneal dialysis; *HD* hemodialysis; *Tx* transplantation; *RRT* renal replacement therapy; *nPNA* normalized protein nitrogen appearance

### Primary and secondary outcomes

**Fig. 3:**
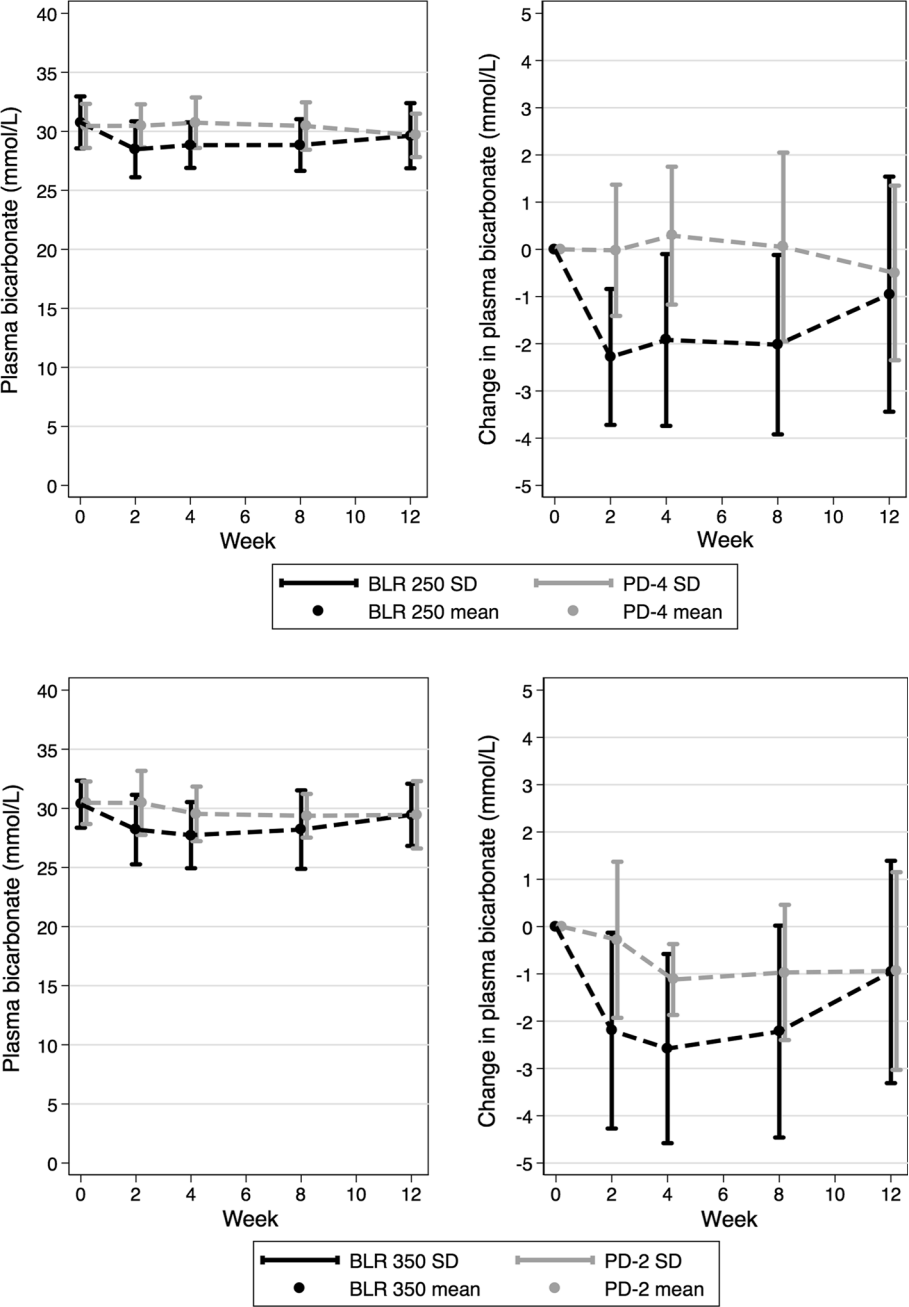
Plasma bicarbonate concentrations and change from baseline in the BLR 250 (*top panel*) and BLR 350 trials (*bottom panel*)

**Fig. 4:**
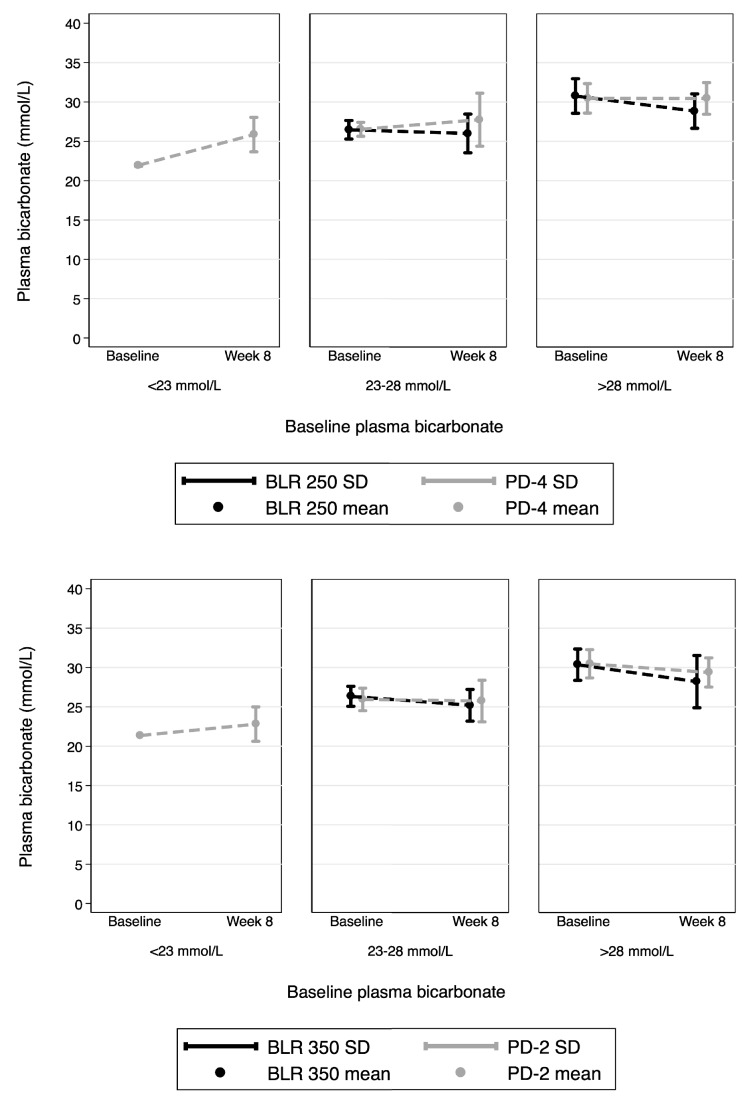
Plasma bicarbonate concentrations and change from baseline in the BLR 250 (*top panel*) and BLR 350 trials (*bottom panel*), stratified by baseline plasma bicarbonate

Table 3 shows estimation results for the primary and secondary outcomes. In both the ITT and PP analyses, both BLR 250 and BLR 350 were non-inferior to the standard PDFs in terms of pCcr and UF volume. There was no difference statistically significant difference in pCurea between investigative and standard PDFs. However, both BLR 250 and BLR 350 demonstrated a significant decrease in plasma bicarbonate concentration compared with the standard solutions, having the mean (95% confidence interval) change of 1.94 (−2.57, −1.31) mmol/L and 1.29 (−1.95, −0.63) mmol/L, respectively. Overall changes in plasma bicarbonate are illustrated in Figs. 3 and 4.

**Table 3:** Estimates (adjusted difference in means) from the analysis of the primary outcomes (peritoneal creatinine clearance in L/week/1.73 m^2^, ultrafiltration volume in L/day) and the secondary outcomes (peritoneal Kt/V_urea_, plasma [HCO_3_^−^] in mmol/L)

Comparison	Outcome	Study population subset	Estimate	Standard deviation	*p* value	95% confidence interval	
						Lower limit	Upper limit
BLR250 vs. Dianeal^**®**^ PD-4	Peritoneal creatinine clearance	PP	−0.39	0.99	*p* = 0.699	−2.36	1.59
BLR250 vs. Dianeal^**®**^ PD-4	Peritoneal creatinine clearance	ITT	−0.46	0.98	*p* = 0.643	−2.41	1.50
BLR250 vs. Dianeal^**®**^ PD-4	Ultrafiltration volume	PP	0.075	0.047	*p* = 0.115	−0.019	0.168
BLR250 vs. Dianeal^**®**^ PD-4	Ultrafiltration volume	ITT	0.071	0.047	*p* = 0.131	−0.022	0.165
BLR350 vs. Dianeal^**®**^ PD-2	Peritoneal creatinine clearance	PP	−0.12	0.84	*p* = 0.890	−1.78	1.55
BLR350 vs. Dianeal^**®**^ PD-2	Peritoneal creatinine clearance	ITT	−0.40	0.81	*p* = 0.625	−2.00	1.21
BLR350 vs. Dianeal^**®**^ PD-2	Ultrafiltration volume	PP	0.125	0.051	*p* = 0.017	0.023	0.227
BLR350 vs. Dianeal^**®**^ PD-2	Ultrafiltration volume	ITT	0.127	0.050	*p* = 0.012	0.029	0.226
BLR250 vs. Dianeal^**®**^ PD-4	Peritoneal Kt/V_urea_	ITT	−0.01	0.03	*p* = 0.822	−0.08	0.06
BLR250 vs. Dianeal^**®**^ PD-4	Plasma [HCO_3_^−^]	ITT	−1.94	0.32	*p* < 0.001	−2.57	−1.31
BLR350 vs. Dianeal^**®**^ PD-2	Peritoneal Kt/V_urea_	ITT	0.03	0.03	*p* = 0.187	−0.02	0.09
BLR350 vs. Dianeal^**®**^ PD-2	Plasma [HCO_3_^−^]	ITT	−1.29	0.33	*p* < 0.001	−1.95	−0.63

There were corresponding changes in plasma pH in those receiving investigative PDFs, which was thought to be due to the observed decreases in plasma bicarbonate concentration, since there were no statistically significant accompanying changes in pCO_2_ (data not shown). In the BLR250 group, pH was 7.40 at baseline, but was significantly lower between 7.38 and 7.39 during the study treatment period (*p* < 0.05). There was no change in the PD-4 group. In the BLR350 group, pH was 7.39 at baseline, but was significantly lower between 7.37 and 7.38 during the study treatment period (*p* < 0.05). There was no change in the PD-2 group.

Table 4 shows changes over time in the plasma bicarbonate concentration for the investigative and standard PDFs, stratified by baseline values. Of note, both of the investigative solutions demonstrated a significant and prominent correction of plasma bicarbonate in those with elevated baseline concentrations, improving them to the normal range without the development of acidosis.

**Table 4:** Changes over time in plasma bicarbonate concentration in mmol/L, by peritoneal dialysis fluid, and stratified by baseline values

Treatment group	Parameter	Baseline period	Study treatment period			Follow-up period
		Week 0	Week 2	Week 4	Week 8	Week 12
		Mean ± SD	Mean ± SD	Mean ± SD	Mean ± SD	Mean ± SD
Baseline plasma [HCO_3_-] >28 mmol/L						
BLR250	Plasma [HCO_3_^−^]	30.76 ± 2.20	28.48 ± 2.37	28.83 ± 1.93	28.84 ± 2.19	29.63 ± 2.76
Dianeal^**®**^ PD-4		30.46 ± 1.87	30.47 ± 1.82	30.73 ± 2.14	30.45 ± 2.01	29.66 ± 1.84
*p* value		0.544	<0.001	<0.001	0.004	0.951
BLR250	Change plasma [HCO_3_^−^] from baseline	–	−2.28 ± 1.44	−1.92 ± 1.82	−2.02 ± 1.90	−0.95 ± 2.49
Dianeal^**®**^ PD-4		–	−0.02 ± 1.39	0.29 ± 1.46	0.05 ± 2.00	−0.50 ± 1.85
*p* value		–	*p* < 0.001	*p* < 0.001	*p* < 0.001	*p* = 0.410
BLR350	Plasma [HCO_3_^−^]	30.35 ± 1.99	28.20 ± 2.94	27.73 ± 2.80	28.20 ± 3.32	29.45 ± 2.63
Dianeal^**®**^ PD-2		30.47 ± 1.80	30.46 ± 2.71	29.53 ± 2.31	29.37 ± 1.85	29.34 ± 2.85
*p* value		*p* = 0.819	*p* = 0.006	*p* = 0.017	*p* = 0.140	*p* = 0.891
BLR350	Change plasma [HCO_3_^−^] from baseline	–	−2.20±2.07	−2.58 ± 2.00	−2.22 ± 2.24	−0.96 ± 2.35
Dianeal^**®**^ PD-2		–	−0.28±1.65	−1.12 ± .75	−0.97 ± 1.43	−0.94 ± 2.09
*p* value		–	*p* = 0.001	*p* = 0.009	*p* = 0.027	*p* = 0.971
Baseline plasma [HCO_3_−] 23–28 mmol/L						
BLR250	Plasma [HCO_3_^−^]	26.46 ± 1.18	25.28 ± 1.60	25.89 ± 1.62	26.00 ± 2.46	27.28 ± 2.10
Dianeal^**®**^ PD-4		26.52 ± 0.89	27.45 ± 2.49	27.28 ± 3.11	27.75 ± 3.37	27.04 ± 3.10
*p* value		*p* = 0.912	*p* = 0.027	*p* = 0.215	*p* = 0.237	*p* = 0.854
BLR250	Change plasma [HCO_3_^−^] from baseline	–	−1.18 ± 0.98	−0.57 ± 1.10	*−*1.06 ± 1.96	0.47 ± 1.57
Dianeal^**®**^ PD-4		–	0.94 ± 2.22	0.88 ± 2.82	1.24 ± 2.90	0.56 ± 2.58
*p* value		–	*p* = 0.017	*p* = 0.188	*p* = 0.074	*p* = 0.931
BLR350	Plasma [HCO_3_^−^]	26.33 ± 1.27	25.79 ± 2.11	25.13 ± 2.11	25.20 ± 2.02	27.18 ± 1.62
Dianeal^**®**^ PD-2		25.94 ± 1.43	26.61 ± 1.64	26.32 ± 2.33	25.74 ± 2.64	26.80 ± 2.70
*p* value		*p* = 0.355	*p* = 0.158	*p* = 0.093	*p* = 0.459	*p* = 0.582
BLR350	Change plasma [HCO_3_^−^] from baseline	–	−0.54 ± 1.81	−1.27 ± 2.08	−1.13 ± 1.50	0.79 ± 1.83
Dianeal^**®**^ PD-2		–	−0.67 ± 1.23	0.29 ± 2.24	−0.30 ± 2.06	0.76 ± 2.03
*p* value		–	*p* = 0.013	*p* = 0.026	*p* = 0.140	*p* = 0.966
Baseline plasma [HCO_3_^−^] <23 mmol/L						
BLR250	Plasma [HCO_3_^−^]	–	–	–	–	–
Dianeal^**®**^ PD-4		21.95 ± 0.14	23.95 ± 0.21	23.40	25.85 ± 2.19	25.20 ± 1.41
*p* value		–	–	–	–	–
BLR250	Change plasma [HCO_3_^−^] from baseline	–	–	–	–	–
Dianeal^**®**^ PD-4		–	2.00 ± 0.35	1.55	3.90 ± 2.05	3.25 ± 1.27
*p* value		–	–	–	–	–
BLR350	Plasma [HCO_3_^−^]	–	–	–	–	–
Dianeal^**®**^ PD-2		21.35	23.30	22.70	22.80	24.90
*p* value		–	–	–	–	–
BLR350	Change plasma [HCO_3_^−^] from baseline	–	–	–	–	–
Dianeal^**®**^ PD-2		–	1.95	1.35	1.45	3.55
*p* value		–	–	–	–	–

### Other outcomes

Formal safety evaluations were performed for regulatory approval of BLR250 and BLR350, according to advice from the Japanese Pharmaceuticals and Medical Devices Agency (https://www.pmda.go.jp/english/). For this analysis, the safety data set was defined as subjects that were randomly assigned to either treatment group, that were treated with the assigned study drug at least once, and whose post-treatment data were available. Adverse events (AEs) that occurred during the study treatment period and the follow-up period were defined and coded by the Medical Dictionary for Regulatory Activities (MedDRA) ver. 7.0.

No deaths were observed either in the BLR250 group or in the Dianeal^**®**^ PD-4 group. No clinically particularly notable AEs occurred in either group. AEs as defined by the MedDRA ver. 7.0 occurred at a percentage of 92.0% (46/50 subjects) and 85.5% (47/55 subjects) in the BLR250 group and the Dianeal^**®**^ PD-4 group, respectively. There was no statistically significant difference in occurrence rate between groups (Fisher’s exact probability test, *p* = 0.366).

No deaths were reported from either the BLR350 group or the Dianeal^**®**^ PD-2 group. No clinically particularly notable AEs occurred in the BLR350 group, while in the Dianeal^**®**^ PD-2 group, an adverse event from which recovery was unlikely, left central retinal vein occlusion (as reported in the care report form), occurred. AEs as defined by the MedDRA ver. 7.0 occurred during the study treatment period and the follow-up period at a percentage of 88.0% (225 events in 44/50 subjects) and 85.2% (246 events in 46/54 subjects) in the BLR350 group and the Dianeal^**®**^ PD-2 group, respectively. There was no statistically significant difference in occurrence rate between groups (Fisher’s exact probability test, *p* = 0.778).

A full description of adverse events is provided in Appendix 1, as on-line supplementary material.

## Discussion

In this study, we demonstrated non-inferiority of two new investigative PDFs to the standard PDF with respect to the primary endpoints and the main efficacy secondary endpoint: the primary endpoints being pCcr (l/week/1.73 m^2^) and UF volume (l/day), with priority assigned to pCcr and the main efficacy secondary endpoint pCurea (Kt/V). Although non-inferiority is not by definition equivalency, there are unlikely to be any clinical differences in the fundamental functionality of the new investigative PDFs compared with the standard PDFs. This proven functionality will allow Japanese patients’ access to highly biocompatible PDFs, with lower lactate concentration and improved biocompatibility [16]. The investigative PDFs in this study use a mixture of both lactate and bicarbonate as buffer, which has been shown previously to optimize peritoneal host defense parameters in comparison to standard lactate-only PDFs [17–19] and to reduce inflammatory markers in peritoneal effluent [20–23]. The mixture also allows for a normal pH without the risk of intracellular acidosis from an overly high paCO_2_, which is unavoidable if one uses a high concentration of bicarbonate alone as a buffer [19, 24–26].

Importantly, we also demonstrated a significant improvement in plasma bicarbonate concentrations to the normal range during treatment with BLR 250/350. In those with metabolic alkalosis due to over-correction of metabolic acidosis, improvement was confirmed by 2 weeks, with no subsequent decrease in plasma bicarbonate concentrations after that time. In those with normal or low serum bicarbonate, there was no statistically significant change in plasma bicarbonate, or difference in effect between standard solutions and BLR 250/350.

**Fig. 5:**
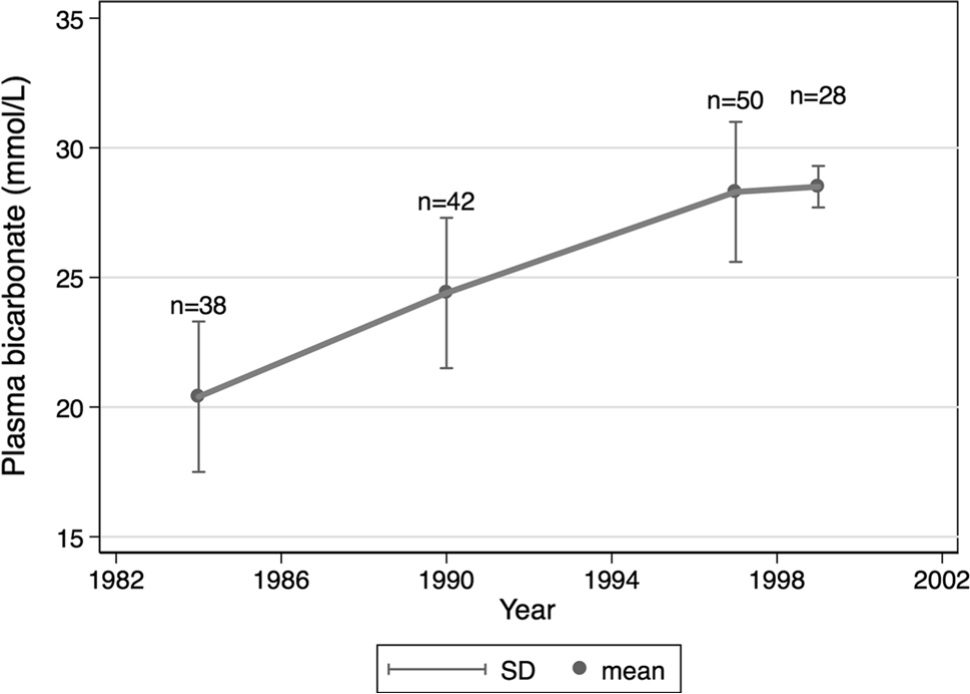
Temporal changes in bicarbonate concentration in continuous ambulatory peritoneal dialysis (PD) patients in Japanese regulatory clinical trials of PD fluids [14, 15, 27, 28]

This feature of BLR 250/350 is clinically important. Metabolic alkalosis seems to occur more frequently in Japanese PD patients, and alkalaemia can been observed to be slowly increasing over time in previously reported trials of PDFs (Fig. 5) [14, 15, 27, 28]. The current alkaline content (40 mmol/L) in the standard PDFs is considered the leading contributor to this situation, along with the frequent use of calcium carbonate as a phosphate binder, and exacerbated by the alkaline food-oriented (e.g. fruits and vegetables) life style of Japanese people. This latter point is supported by the relatively low nPNA in this patient population compared with European or Western cohorts (Table 2).

There is an increasing body of data in the literature suggesting adverse clinical outcomes with metabolic alkalosis. Acute alkalosis during hemodialysis results in increased neuromuscular excitability, reduced cerebral blood flow, respiratory suppression, intra-dialytic hypotension, and cardiac arrhythmias with prolongation of the corrected (QTc) interval on ECGs (mediated in part by associated drops in serum potassium and ionized calcium) [29–34]. Acute alkalosis also increases binding of oxygen to hemoglobin, preventing the release of oxygen to peripheral tissues [35]. Recently, analyses from the Dialysis Outcomes and Practice Patterns Study (DOPPS) showed that long-term mild chronic alkalosis was associated with an increased risk of death in HD patients [adjusted HR, 1.08 per 4 mmol/L higher (95% CI 1.01–1.15); HR for dialysate bicarbonate ≥38 vs 33–37 mmol/L, 1.07 (95% CI 0.97–1.19)] [36]. This important association was stronger in patients with longer dialysis vintage, suggesting cumulative harm over extended exposure, a finding of direct relevance to Japanese dialysis populations given their longevity. Overall, it seems appropriate to avoid chronic alkalosis from over-correction by the high alkaline content of PDF, and individualize PDFs to provide appropriate alkaline content as appropriate [37, 38].

Another important effect of metabolic alkalosis may be enhanced precipitation of calcium phosphate in soft tissues, including vessel walls. Periarticular calcification is associated with alkalosis in HD patients [39–41] and has been reported in alkalotic PD patients treated with a 40 mmol/L lactate-based PDF [42]. This suggests a mechanism by which metabolic alkalosis may contribute to the pathogenesis of cardiovascular disease, especially in the presence of high serum calcium or phosphate levels. This is supported by the DOPPS study above: cardiovascular hospitalization was significantly increased with high dialysate bicarbonate, and cardiovascular mortality showed a corresponding non-significant trend to increase [36].

In summary, this trial demonstrated the suitability of BLR 250/350 for correcting metabolic acidosis in Japanese PD patients. The corrective action of these new PDFs with respect to over-correction of metabolic acidosis could mitigate cardiovascular morbidity and mortality, and is a step towards improved biocompatibility of PDFs. Currently, advantageous features of both BLR250 and BLR350, alone or in combination, may offer a closer to ideal PDF for the Japanese PD population.

## Electronic supplementary material

Below is the link to the electronic supplementary material.

Supplementary material 1 (DOCX 109 kb)

Supplementary material 2 (DOCX 23 kb)
