# Supplementary material for: A new peritoneal dialysis fluid for Japanese patients: a randomized non-inferiority clinical trial of safety and efficacy
Source: Clin Exp Nephrol. 2016 Oct 25;21(5):895–907. doi: 10.1007/s10157-016-1346-9 (PMC5648742; doi:10.1007/s10157-016-1346-9)
Supplement: Supplementary file 1 — Supplementary material 1 (DOCX 109 kb) [file 10157_2016_1346_MOESM1_ESM.docx]

Appendix 1

Unless otherwise indicated, preferred terms (PT) of MedDRA (ver. 7.0) are used in the following summaries.

Brief Summary of Adverse Events - BLR250

No deaths were observed either in the BLR250 group or in the Dianeal PD-4 group. No clinically particularly notable adverse events occurred in either group.

Adverse events (AEs) occurred during the study treatment period and the follow-up period at a percentage of 92.0% (46/50 subjects) and 85.5% (47/55 subjects) in the BLR250 group and the Dianeal PD-4 group, respectively. There was no statistically significant difference in occurrence rate between groups (Fisher’s exact probability test, p=0.366). AEs frequently observed in the BLR250 group included edema peripheral (32.0%, 19 events in 16 subjects), C-reactive protein increased (12.0%, 7 events in 6 subjects), malaise (12.0%, 6 events in 6 subjects), procedural complication (10.0%, 6 events in 5 subjects), weight increased (10.0%, 5 events in 5 subjects), and fluid retention (10.0%, 5 events in 5 subjects). AEs frequently observed in the Dianeal PD-4 group included edema peripheral (25.5 %, 17 events in 14 subjects), C-reactive protein increased (20.0 %, 15 events in 11 subjects), catheter site infection (18.2%, 11 events in 10 subjects), malaise (16.4%, 9 events in 9 subjects), blood pressure increased (12.7%, 9 events in 7 subjects), and fluid retention (12.7%, 8 events in 7 subjects).

Adverse drug reactions (ADRs) occurred during the study treatment period and the follow-up period at a percentage of 26.0 % (13/50 subjects) and 29.1 % (16/55 subjects) in the BLR250 group and the Dianeal PD-4 group, respectively. There was no statistically significant difference in occurrence rate between groups (Fisher’s exact probability test, p=0.828). ADRs frequently observed in the BLR250 group included edema peripheral (12.0%, 7 events in 6 subjects), weight increased (6.0%, 3 events in 3 subjects), and fluid retention (6.0%, 3 events in 3 subjects). ADRs frequently observed in the Dianeal PD-4 group included fluid retention (9.1%, 5 events in 5 subjects), edema peripheral (5.5%, 5 events in 3 subjects), and pCO_2_ increased (5.5%, 4 events in 3 subjects).

The severity of ADRs reported from the BLR250 group was mild in 22% (11/50 subjects, 21 events), moderate in 14.0% (7/50 subjects, 10 events), and severe in 2.0% (1/50 subjects, 4 events). The 4 events considered severe were all observed in the same subject, which were cardiomegaly, weight increased, fluid retention, and hypertension. The severity of ADRs reported from the Dianeal PD-4 group was mild in 21.8 % (12/55 subjects, 28 events), moderate in 14.5 % (8/55 subjects, 25 events), and severe in 1.8 % (1/55 subjects, 1 event). The ADR considered severe was 1 event of fluid retention.

Of the 385 AEs of which relationship to the administered study drug was ruled out (171 and 214 events in the BLR250 and Dianeal PD-4 groups, respectively), there were 2 events of peritonitis and 1 event each of hypoglycemia, hypoglycaemia unawareness, convulsion, and aortic aneurysm in the BLR250 group and 1 event each of infection, increased C-reactive protein, hypoxia, congestive heart failure, and pleural effusion in the Dianeal PD-4 group.

There were 15 and 9 reports of serious AEs from 4 and 3 subjects of the BLR250 group and Dianeal PD-4 group, respectively. Serious AEs reported from the BLR250 group were (by verbatim terms in CRFs) 1 event each of phlegmon in the right leg, left suprapatellar bursitis, Baker’s cyst in the left knew, left knee swelling and aggravated atheroma in the gluteal region, abdominal pain, fever, CRP increased, and viral enterocolitis. All these events were judged Not Related to the study drug.

Serious AEs observed in the Dianeal PD-4 group were (by verbatim terms in CRFs) 1 event each of aggravated numbness in the right hand, white blood cells increased (28500/μL), CRP increased, systemic infection, hypoxemia, acute heart failure, serum osmolarity increased, heart failure, and angina unstable. All these events were judged Not Related to the study drug.

AEs that led to study discontinuation other than those reported as serious AEs comprised 5 events in 2 subjects in the BLR250 group and 4 events in 4 subjects in the Dianeal PD-4 group. Those events observed in the BLR250 group were (by verbatim terms in CRFs) 1 event each of thoracic aortic aneurysm, inadequate ultrafiltration, hypertension aggravated, weight increased, and cardiothoracic ratio increased as detected by chest X-ray. The thoracic aortic aneurysm was judged Not Related, while the other 4 events were deemed Probably Related.

Those events observed in the Dianeal PD-4 group were (as reported in CRFs) 1 event each of pleural effusion aggravated, cardiac failure congestive due to inadequate ultrafiltration, indefinite complaint, and inadequate ultrafiltration. The inadequate ultrafiltration was judged Probably Related, and the other events judged Not Related.

Brief Summary of Adverse Events - BLR350

No deaths were reported from either the BLR350 group or the Dianeal PD-2 group. There were no clinically particularly notable adverse events in the BLR350 group, while in the Dianeal PD-2 group an adverse event from which recovery was unlikely, left central retinal vein occlusion (as reported in the CRF), occurred.

Adverse events occurred during the study treatment period and the follow-up period at a percentage of 88.0 % (225 events in 44/50 subjects) and 85.2 % (246 events in 46/54 subjects) in the BLR350 group and the Dianeal PD-2 group, respectively. There was no statistically significant difference in occurrence rate between groups (Fisher’s exact probability test, p=0.778). Adverse events frequently observed in the BLR350 group included oedema peripheral (28.0 %, 15 events in 14 subjects), weight increased (22.0%, 13 events in 11 subjects), C-reactive protein increased (18.0 %, 10 events in 9 subjects), myospasm (16.0%, 10 events in 8 subjects), fluid retention and facial oedema (both 14.0 %, 7 events in 7 subjects), cardiomegaly and blood pressure increased (both 12.0%, 6 events in 6 subjects), malaise (10.0%, 7 events in 5 subjects), and nasopharyngitis (10.0%, 5 events in 5 subjects). In the Dianeal PD-2 group, frequently observed AEs included malaise, oedema peripheral, and blood pressure increased (all, 16.7 %, 9 events in 9 subjects), fluid retention (14.8%, 8 events in 8 subjects), nasopharyngitis (11.1%, 7 events in 6 subjects), and diarrhea and weight increased (both 11.1%, 6 events in 6 subjects).

Adverse drug reactions occurred during the study treatment period and the follow-up period at a percentage of 52.0 % (74 events in 26/50 subjects) and 35.2 % (67 events in 19/54 subjects) in the BLR350 group and the Dianeal PD-2 group, respectively. There was no statistically significant difference in occurrence rate between groups (Fisher’s exact probability test, p=0.113). Adverse drug reactions frequently observed in the BLR350 group included oedema peripheral (22.0 %, 11 events in 11 subjects), body weight increased (14.0%, 7 events in 7 subjects), fluid retention (12.0%, 6 events in 6 subjects), facial oedema (10.0%, 5 events in 5 subjects), and cardiomegaly and C-reactive protein increased (both, 8.0%, 4 events in 4 subjects). In the Dianeal PD-2 group, frequently observed ADRs included fluid retention (11.1 %, 6 events in 6 subjects), oedema peripheral and body weight increased (both 7.4 %, 4 events in 4 subjects), and cardiomegaly and malaise (both, 5.6%, 3 events in 3 subjects).

The severity of ADRs reported from the BLR350 group was mild in 46% (23/50 subjects, 55 events), moderate in 18.0% (9/50 subjects, 15 events), and severe in 4.0% (2/20 subjects, 4 events). The ADRs rated severe comprised peritonitis reported from 1 subject and oedema peripheral, weight increased, and facial oedema observed in 1 other subject. The severity of ADRs reported from the Dianeal PD-2 group was mild in 24.1 % (13/54 subjects, 25 events), moderate in 22.2 % (12/54 subjects, 30 events), and severe in 5.6 % (3/54 subjects, 12 event). The 12 events rated severe comprised corneal oedema, eye pain, glaucoma, retinal vein occlusion, misty vision, corneal disorder, and intraocular pressure increased observed in 1 subject, and lower abdominal pain, upper abdominal pain, nausea, and vomiting in 1 other subject, and weight increased in 1 still other subject.

Of the adverse events of which relationship to the administered study drug was ruled out counted 330 events (151 and 179 events in the BLR350 and Dianeal PD-2 groups, respectively), there were 1 event each of peritonitis and infusion site pain in the BLR350 group and 3 events of peritonitis and 1 event each of device failure, blood albumin decreased, blood potassium decreased, C-reactive protein increased, and protein total decreased.

There were 3 serious adverse events in 3 subjects of the BLR350 group and 19 serious adverse events in 6 subjects of the Dianeal PD-2 group. Those serious events observed in the BLR350 group were (as reported in CRFs) 1 event each of weight increased, double vision, and CAPD peritonitis. Cases of weight increased and double vision were rated Unrelated to the study drug, and CAPD peritonitis rated Probably Related.

Serious adverse events that occurred in the Dianeal PD-2 group comprised (as reported in CRFs) 2 events of peritonitis and 1 event each of “crack in the CAPD catheter,” “natural rupture of Tenckhoff Catheter,” “misty vision,” “left central retinal vein occlusion,” “left eye pain,” “left neovascular glaucoma,” “increased left intraocular pressure,” “lower limb oedema,” “increased cardiothoracic ratio,” “aggravated nephrogenic anemia,” “malaise,” “symptoms of uremia,” “cloudy effluent,” “decreased Alb,” “decreased K,” “increased CRP,” and “decreased TP.”

“Double vision,” “left central retinal vein occlusion,” “left eye pain,” “left neovascular glaucoma,” “increased left intraocular pressure,” “lower limb oedema,” “increased cardiothoracic ratio,” “aggravated nephrogenic anemia,” “malaise,” and “symptoms of uremia were rated Probably Related to the study drug.

Adverse events that led to study discontinuation other than those reported as serious adverse events comprised 5 events in 2 subjects in the BLR350 group and 1 events in 4 subjects in the Dianeal PD-2 group. Those events observed in the BLR350 group were (as reported in CRFs) 1 event each of CAPD-related peritonitis, pain during dwell, lower limb oedema, weight increased, and facial oedema. CAPD-related peritonitis and pain during dwell were judged Unrelated. Lower-limb oedema, weight increased, and facial oedema were judged Probably Related. Those events observed in the Dianeal PD-2 group were (as reported in CRFs) 1 event each of pain in lower abdomen, gastric pain, and nausea, and vomiting. All these were rated “Probably Related” to the study drug.
