# Supplementary material for: A new peritoneal dialysis fluid for Japanese patients: a randomized non-inferiority clinical trial of safety and efficacy
Source: Clin Exp Nephrol. 2016 Oct 25;21(5):895–907. doi: 10.1007/s10157-016-1346-9 (PMC5648742; doi:10.1007/s10157-016-1346-9)
Supplement: Supplementary file 2 — Supplementary material 2 (DOCX 23 kb) [file 10157_2016_1346_MOESM2_ESM.docx]

# Appendix 2

Medical institutions for BLR 250 trial

| No. | Medical institutions | Investigators |
| --- | --- | --- |
| 1 | Asahikawa Red Cross Hospital | 1. Izumi Yamachi  2. Atsushi Wada |
| 2 | Sendai Social Insurance Hospital | Yoshio Taguma |
| 3 | Tokyo Jikei-kai Medical School Hospital | Tatsuo Hosoya |
| 4 | Tokyo Jikei-kai Medical School Kashiwa Hospital | 1. Yasuo Kimura  2. Makoto Ogura |
| 6 | Mitsui Memorial Hospital | Tokuichiro Sugimoto |
| 7 | Nihon University Itabashi Hospital | Kouichi Matsumoto |
| 8 | St. Marianna University Scahool of Medicine Hospital | Gouichi Ogimoto |
| 9 | Hospital Affiliating with Kanagawa Prefecture Nursing School | Yoshindo Kawaguchi |
| 10 | Showa University Fujigaoka Hospital | Terukuni Ideura |
| 12 | Aichi Medical University Hospital | Arao Futenma |
| 13 | Chukyo Hospital | Tomio Yamamoto |
| 14 | Clinic affiliating with Inoue Hospital | Yoshihiro Tsujimoto |
| 15 | Kinki University School of Medicine Hospital | Hirofumi Hasegawa |
| 16 | Osaka Koseinenkin Hospital | Masamitsu Fujii |
| 17 | Hiroshima University Hospital | Noriaki Yorioka |
| 18 | Tokushima Red Cross Hospital | Akihiro Sakata |
| 19 | Saiseikai Yahata General Hospital | Masahiko Nakamoto |
| 20* | Toho University School of Medicine Omori Hosital | Akira Hasegawa |
| 21 | Shizuoka Genaral Hospital | Norko Mori |
| 22* | Amjyo Kyoritsu Clinic | Hachiro Senoh |
| 23* | Kaikoukai Central Clinic | Yasumasa Kawade |
| 24 | Shirasagi Clinic | Tsuyoshi Izumotani |
| 25 | Osaka City University School of Medicine Hospital | Yoshiaki Takemoto |
| 26 | Teine Keijinkai Hospital | Hideki Takizawa |
| 27 | Tokai University School of Medicine Hospital | Akira Saito |
| 28 | Tokuyama Central Hospital | Shigeaki Hayashida |
| 29 | Hakodate Goryoukaku Hospital | Katsuo Suzuki |
| 30 | Kawasaki Medical School Hospital | Tamaki Sasaki |
| 31 | Saiseikai Central Hospital | Satoru Kuriyama |
| 32 | Tokyo Kyosai Hospital | Hiroyuki Tamura |
| 33 | Kumamoto Central Hospital | Kenji Arizono |
| *There is an agreement of clinical trial with the institute, but there is no case. | | |

Note **:**  Affiliation of each doctor is at the time of clinical study.

Medical institutions for BLR 350 trial

Note **:**  Affiliation of each doctor is at the time of clinical study.

| No. | Medical institutions | Investigators |
| --- | --- | --- |
| 1 | Obihiro-Kosei General Hospital | Takahiro Nishitani |
| 2 | Caress Alliance Nikko-Kinen Hospital | Noritomo Itami |
| 3 | Yamagata University School of Medical Hospital | Tsuneo Konta |
| 4 | Toride Kyodo General Hospital | Yoshitaka Maeda |
| 5 | Kameda Medical Center | Takahiro Mochizuki |
| 6 | Saitama Medical University Hospital | Hiromichi Suzuki |
| 7 | Saitama Medical University Medical Center | Tetsuya Mitarai |
| 8 | Tokyo Medical University Hospital | Toshiyuki Nakao |
| 9 | Tokyo Jikeikai University Hospital | Tatsuo Hosoya |
| 10 | Tokyo Women’s Medical University Hospital | Takashi Akiba |
| 11 | Juntendo University School of Medical Hospital | Yasuhiko Tomino |
| 12 | Tokyo Women’s Medical University Second Hospital | Tsutomu Sanaka |
| 13* | Showa University Hospital | Tetsuzo Sugisaki |
| 14 | Toranomon Hospital | 1. Shigeko Hara  2. Kenmei Takaichi |
| 15 | Yokosuka Kyosai Hospital | Takao Shoji |
| 16 | Shonan Kamakura General Hospital | Shuzo Kobayashi |
| 17 | Fujita Health University Hospital | Satoshi Sugiyama |
| 18* | Kakegawa City General Hospital | Akira Orihara |
| 19 | Nara Medical University Hospital | Toshihiko Nishino |
| 21 | Okayama Saiseikai General Hospital | Makoto Hiramatsu |
| 22 | Kurashiki Central Hospital | Masaki Fukushima |
| 23 | Akane-kai Tschiya General Hospital | Hideki Kawanishi |
| 24 | Takamatsu Red Cross Hospital | Akira Numata |
| 25 | Kumamoto Central Hospital | 1. Hiroyoshi Fukui  2. Kenji Arizono |
| 26 | Saiseikai Kumamoto Hospital | Hidehisa Soejima |
| 27 | Toranomon Hospital Annex | 1. Shigeko Hara  2. Kenmei Takaichi |
| 28 | Nankai Hospital | Takahisa Kamegawa |
| 29 | St. Luke’s International Hospital | Yasuhiro Komatsu |
| 30 | Tokyo Saiseikai Central Hospital | Satoru Kuriyama |
| 31 | Gifu Prefectural General Medical Center | Hiroshige Ohasi |
| 32 | Hiroshima University Hospital | Noriaki Yorioka |

*There is an agreement of clinical trial with the institute, but there is no case.
